# Breastfeeding: Investing in the Future

**DOI:** 10.1089/bfm.2019.0032

**Published:** 2019-04-12

**Authors:** Bernardo L. Horta

**Affiliations:** Post Graduate Program in Epidemiology, Federal University of Pelotas, Pelotas, Brazil.

The short-term benefits of breastfeeding have been well established. Among infants 0–5 months of age, the risk of all-cause mortality has been estimated to be 14 times higher in nonbreastfed infants compared with exclusively breastfed infants.^1^ A systematic review and meta-analysis reported a lower risk of diarrhea morbidity in infants ≤6 months of age who were exclusively breastfed versus those who were not breastfed (relative risk [RR] = 0.20 [95% confidence interval (CI): 0.13–0.29]).^2^ The risk of morbidity from diarrhea was also lower among infants <6 months of age who were breastfed (RR = 0.46 [0.28–0.78]). Breastfeeding was also associated with reduced risk of diarrhea mortality (RR = 0.23 [0.13–0.42]), with the lowest risk among children <12 months of age. The protective effects of breastfeeding against respiratory infection were also assessed; breastfeeding was associated with reduced risk of morbidity (RR = 0.68 [0.60–0.77]) and mortality (RR = 0.30 [0.16–0.56]) from lower respiratory tract infections.^2^ Possible mechanisms for the beneficial short-term effects of breastfeeding include immunologic factors present in human milk (e.g., antimicrobial and anti-inflammatory factors, digestive enzymes), lower exposure to pathogens, and better nutritional status.

**FIG. 1.**
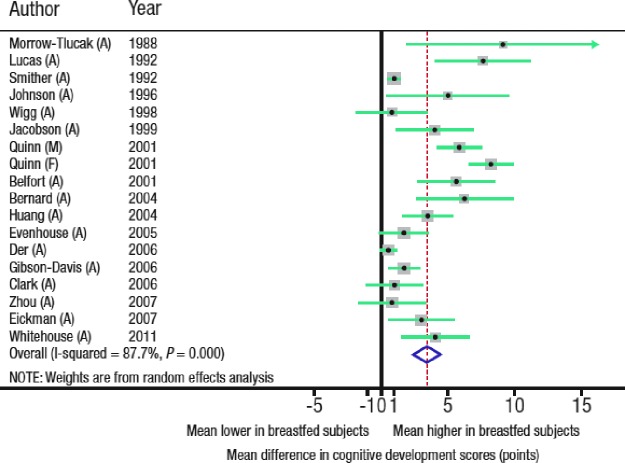
Breastfeeding is positively associated with performance in intelligence tests in childhood and adolescence.^3^

There is increasing evidence that breastfeeding also confers long-term benefits to the infant, not only in health and disease, but also in the development of human capital. A meta-analysis of 17 observational studies evaluating the association between breastfeeding and performance on intelligence tests in childhood and adolescence found that breastfed infants achieved a higher IQ (difference of 3.4 points [95% CI: 2.3–4.6]) compared with nonbreastfed infants (Fig. 1).^3^ The association remained after adjustment for maternal IQ (difference of 2.6 points [1.3–4.0]). The meta-analysis excluded studies that did not control for child stimulation or interaction at home. These results were supported by findings from a cluster-randomized controlled trial (Promotion of Breastfeeding Intervention Trial [PROBIT]) that assessed the effects of the Baby-friendly Hospital Initiative on breastfeeding duration, exclusivity, and outcomes.^4^ Infants randomized to breastfeeding promotion were breastfed longer and had a 7.5-point higher verbal IQ score at 6.5 years of age versus children in the control arm. The association between breastfeeding and improved performance on intelligence tests may be related to residual confounding by socioeconomic status. However, an association between breastfeeding and IQ was observed in the Brazilian Pelotas cohort study, a population in which there is no association between family income and breastfeeding, suggesting that residual confounding due to socioeconomic status was unlikely.^5^

**FIG. 2.**
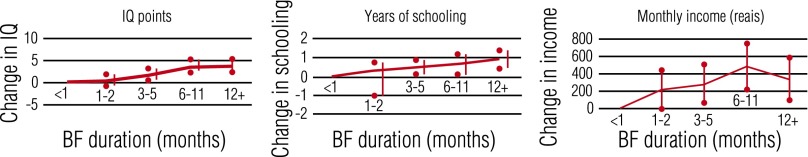
Association between breastfeeding duration and IQ, years of schooling, and monthly income at 30 years of age.^6^ Analyses adjusted for 11 confounding factors. BF, breastfed.

To determine whether the effects of breastfeeding on IQ were long lasting, data from the Pelotas cohort study were used to assess the impact of breastfeeding in participants 30 years of age.^6^ Among the 3,493 participants for which data were available, duration of breastfeeding was positively associated with IQ, educational attainment, and income (Fig. 2). Mediation analysis showed that IQ explained 72% of the effect of breastfeeding on income. After controlling for an allele score used to predict schooling, the estimates of the association between breastfeeding duration and IQ were similar to those seen after controlling for other confounding variables, suggesting the association was probably not caused by residual confounding due to genetic factors.

Data from the British ALSPAC cohort study were used to assess the economic impact of breastfeeding.^7^ There was a positive association between breastfeeding and achieving higher educational attainment at 16 years of age (defined as having five General Certificate of Secondary Education [GCSE] grades of C or higher). Health economic models estimated that the average lifetime income was expected to be £67,535 higher for children achieving ≥5 passing GCSE grades compared with those achieving <5 passing GCSE grades. Incorporating the impact of breastfeeding on educational attainment translates into an expected lifetime income £4,208 higher for children who were breastfed for <6 months and £8,799 higher for children who were breastfed for ≥6 months compared with children who were never breastfed (Table 1).

**Table 1.** Effects of Breastfeeding on Income

	*Income in £ (95% CI)*
Expected lifetime income (never breastfed)	473,255
Expected lifetime income (breastfed <6 months)	477,463 (475,193–479,962)
Benefit per child	4,208 (1,938–6,708)
Expected lifetime income (breastfed ≥6 months)	482,054 (479,274–484,782)
Benefit per child	8,799 (6,019–11,528)

CI, confidence interval.

Source: Straub et al., 2016.^7^

There is also evidence to suggest breastfeeding may provide long-term health benefits to the mother. A meta-analysis of 98 studies reported a 20% reduction in breast cancer risk in women who breastfed versus those who never breastfed (odds ratio [OR] = 0.78 [95% CI: 0.75–0.82]).^8^ Pooled results from 41 studies showed a 30% reduction in ovarian cancer risk in women who breastfed versus those who never breastfed (OR = 0.70 [0.64–0.77]). The effect was attenuated somewhat when the studies were restricted to those with fine adjustment for parity and exclusion of nulliparous women (OR = 0.82 [0.75–0.89]).

In summary, breastfeeding has short-term benefits for child survival and is positively associated with long-term benefits in human capital, including improved performance in intelligence tests, greater academic achievement, and higher income. Possible mechanisms for these long-term benefits may be related to the abundance of long-chain polyunsaturated fatty acids in breast milk, which are important for brain growth and development. Breastfeeding may also facilitate maternal bonding, which may in turn contribute to child development. The health and cognitive benefits associated with breastfeeding reinforce the importance of breastfeeding promotion and may have important health and economic implications.
